# The Risk of Hearing Impairment From Ambient Air Pollution and the Moderating Effect of a Healthy Diet: Findings From the United Kingdom Biobank

**DOI:** 10.3389/fncel.2022.856124

**Published:** 2022-04-06

**Authors:** Lanlai Yuan, Dankang Li, Yaohua Tian, Yu Sun

**Affiliations:** ^1^Department of Otorhinolaryngology, Union Hospital, Tongji Medical College, Huazhong University of Science and Technology, Wuhan, China; ^2^Department of Maternal and Child Health, School of Public Health, Tongji Medical College, Huazhong University of Science and Technology, Wuhan, China; ^3^Ministry of Education Key Laboratory of Environment and Health, State Key Laboratory of Environmental Health (Incubating), School of Public Health, Tongji Medical College, Huazhong University of Science and Technology, Wuhan, China

**Keywords:** hearing impairment, air pollution, digit triplet test (DTT), United Kingdom Biobank (UKB), healthy diet

## Abstract

The link between hearing impairment and air pollution has not been established, and the moderating effect of a healthy diet has never been investigated before. The purpose of this study was to investigate the association between air pollution and hearing impairment in British adults aged 37–73 years, and whether the association was modified by a healthy diet. We performed a cross-sectional population-based study with 158,811 participants who provided data from United Kingdom Biobank. A multivariate logistic regression model was used to investigate the link between air pollution and hearing impairment. Subgroup and effect modification analyses were carried out according to healthy diet scores, gender, and age. In the fully adjusted model, we found that exposure to PM_10_, NO_X_, and NO_2_ was associated with hearing impairment [PM_10_: odds ratio (OR) = 1.15, 95% confidence interval (95% CI) 1.02–1.30, *P* = 0.023; NO_X_: OR = 1.02, 95% CI 1.00–1.03, *P* = 0.040; NO_2_: OR = 1.03, 95% CI 1.01–1.06, *P* = 0.044], while PM_2.5_ and PM_2.5_ absorbance did not show similar associations. We discovered an interactive effect of age and air pollution on hearing impairment, but a healthy diet did not. The findings suggested that exposure to PM_10_, NO_X_ and NO_2_ was linked to hearing impairment in British adults, whereas PM_2.5_ and PM_2.5_ absorbance did not show similar associations. These may help researchers focus more on the impact of air pollution on hearing impairment and provide a basis for developing effective prevention strategies.

## Introduction

Hearing impairment is one of the most common age-related chronic health problems (Vos et al., 2016). The rate of clinically significant hearing impairment is doubling approximately every decade (Lin et al., 2011; Goman and Lin, 2016). Hearing impairment has been reported to be the second most prevalent disorder and the dominant cause of years lived with disability among global non-infectious diseases (Vos et al., 2016). In contrast with normal hearing adults of the same age, those with hearing impairment have a greater incidence of hospitalization (Genther et al., 2013), death (Contrera et al., 2015), falls (Lin and Ferrucci, 2012), cardiovascular disorders (McKee et al., 2018), depression (Li et al., 2014), and dementia (Lin et al., 2013). Consequently, hearing impairment causes a huge burden on the emotional and physical wellbeing of individuals (Dawes et al., 2014b). It is predicted that one-fifth of the population of the United Kingdom will suffer from hearing impairment by 2035 (Taylor et al., 2020). Accordingly, the key is to prevent hearing impairment. Hearing impairment is caused by a combination of hereditary and environmental factors (Cunningham and Tucci, 2017). The identification of modifiable risk factors is critical to provide the basis for preventive strategies.

Global trends in urbanization and industrialization have led to a growing problem of air pollution (Landrigan, 2017), which has become the main public health issue across the world (Brunekreef and Holgate, 2002). Of note, growing evidence demonstrates that air pollution exposure is not only connected with respiratory disorders, such as lung cancer (Xing et al., 2019), but also with cardiovascular diseases (Lelieveld et al., 2019; Hayes et al., 2020), inflammatory diseases (Chang et al., 2016), diabetes (Strak et al., 2017), and neurodegenerative diseases (Chen et al., 2017). Besides, the main environmental risk factor for human death is air pollution (Gordon et al., 2014). Lately, there have been reports that air pollution may impact hearing health, but available data is limited. A recent study (Tsai et al., 2020) found that participants exposed to fine particulate matter (PM_2.5_: particulate matter ≤ 2.5 μm in diameter) and nitrogen dioxide (NO_2_) had a substantially increased risk of sudden sensorineural hearing loss (SSNHL). Another study (Chang et al., 2020) showed that increased concentrations of NO_2_ were linked to a higher risk of sensorineural hearing loss, while in a nested case-control study (Choi et al., 2019), SSNHL was associated with NO_2_ exposure, but particulate matter with a diameter of 10 μm or less (PM_10_) was not associated with SSNHL. Similarly, another study (Lee et al., 2019) also found no association between PM_10_ and number of SSNHL patient. Although these studies explored the association of air pollution with sensorineural hearing loss, the results remained controversial.

A healthy diet might preserve hearing (Spankovich and Le Prell, 2013; Curhan et al., 2018, 2020), as described by their role in preventing chronic illnesses (Yevenes-Briones et al., 2021). A healthy diet includes multiple components that support antioxidant function and protect against free radical damage (Curhan et al., 2020), thereby regulating oxidative stress and delaying mitochondrial dysfunction (Yevenes-Briones et al., 2021). In addition, a healthy diet might be beneficial to hearing impairment by protecting microvascular and macrovascular damage to cochlear blood flow (Appel et al., 2006; Fung et al., 2008), providing the essential nutrients for an adequate cochlear blood supply (Yevenes-Briones et al., 2021), and reducing inflammation (Neale et al., 2016). According to previous research, dietary patterns could modify the relationship between air pollution and health-related outcomes, such as cardiovascular disease mortality risk (Lim et al., 2019) and cognitive function (Zhu et al., 2022). However, the moderating effect of a healthy diet on the link between hearing impairment and air pollution has not been investigated before. Therefore, in this cross-sectional study, we aimed to explore the link between air pollution and hearing impairment and to analyze whether a healthy diet has moderating effects on this link.

## Materials and Methods

### Study Subjects

The United Kingdom Biobank is an international and accessible data resource^1^ containing data on more than half a million people aged from 37 to 73 years (99.5% were between 40 and 69 years) in England, Scotland, and Wales (Collins, 2012). Adults living within a 25-mile radius of one of 22 Biobank Assessment Centers in the United Kingdom were invited by email to join the United Kingdom Biobank between 2006 and 2010, achieving a response rate of approximately 5.5% (Sudlow et al., 2015). Participants completed a computer touch screen questionnaire (which included questions on topics such as population, health, lifestyle, environment as well as medical history, etc.) and underwent physical measurements, including a hearing test. Written informed consent was signed by all the participants. The research was carried out with the general approval of the National Health Service and the National Research Ethics Service. The subjects of the current study were all those participants for whom data on both air pollution measures and hearing test results were available.

### Hearing Test

The speech-in-noise hearing test (i.e., digit triplet test, DTT) of the United Kingdom Biobank provided participants with 15 groups of English monosyllabic numbers to evaluate the listening thresholds (i.e., signal-to-noise ratio) at different sound levels.^2^ Each ear was examined separately, in the order that the participants were allocated at random. Participants first wore circumaural headphones and selected the most comfortable volume. Then, they started the speech-in-noise hearing test to identify and type the three numbers they had heard by touching the screen interface. The noise level of the subsequent triple would increase if the triplet was correctly recognized; otherwise, it would reduce. The speech reception threshold (SRT) was defined as the signal-to-noise ratio of correctly understanding half of the presented speech. The SRT ranged from −12 to +8 dB, with a lower score representing better performance. Based on the cutoff point established by Dawes et al. (2014b), the better performance ear was chosen for this study, and participants were divided into normal (SRT < −5.5 dB) and hearing impairment (SRT ≥ −5.5 dB) groups.

The DTT shows a very good correlation with the pure tone hearing test (*r* = 0.77) (Jansen et al., 2010), so it can be considered as a measure of hearing impairment (Dawes et al., 2014b). There are some advantages to the DTT, for example, there is no need for a sound booth and the test can be delivered *via* the internet (Moore et al., 2014). The most common hearing complaint is difficulty in hearing over background noise (Pienkowski, 2017), so the speech-in-noise hearing test used to evaluate hearing function represents an ecologically effective as well as objective hearing indicator (Couth et al., 2019).

### Measures of Air Pollution

The air pollution data recorded in the United Kingdom Biobank were from the Small Area Health Statistics Unit,^3^ a part of the BioShaRE-EU Environmental Determinants of Health Project.^4^ The Land Use Regression model was applied to assess air pollution in 2010 by modeling at each residential address of the participants, which was developed as part of the European Study of Cohorts for Air Pollution Effects.^5^ The Land Use Regression model used to calculate the spatial distribution of air pollutants was based on geographic predictors such as traffic, land use, and topography in the geographical information system. In this study, the air pollutants assessed were PM_2.5_, PM_10_, PM_2.5_ absorbance, NO_X_, and NO_2_, of which all were annual average concentrations in μg/m^3^. More details about the air pollution data used in the United Kingdom Biobank are available elsewhere.^6^

### Assessment of Other Variables

Age, gender, ethnicity, educational background, employment, smoking status, and alcohol intake were utilized as baseline data. The ethnic background of participants was divided into six categories: White, Black, Asian, Chinese, Mixed, and other. The educational background was divided into six categories: higher national diploma (HND), national vocational qualification (NVQ), higher national certificate (HNC), or equivalent; A levels or AS levels (including the higher school certificate), or equivalent; O levels (including the school certificate), general certificate of secondary educations (GCSEs), or equivalent; certificate of secondary educations (CSEs), or equivalent; college or university degree; and other professional qualification. Employment status was divided into seven categories: retired; unable to work because of sickness or disability; looking after home and/or family; unemployed; in paid employment or self-employed; student (full-time or part-time); or doing unpaid or voluntary work. Smoking status (Dawes et al., 2014a) was divided into three categories: never-smokers, current and former smokers. Alcohol consumption frequency was divided into five categories: daily or almost daily; three or four times a week; once or twice a week; occasional drinking; and never. Body mass index (BMI) was categorized as obese (BMI ≥ 30), overweight (25 ≤ BMI < 30), normal weight (18.5 ≤ BMI < 25), and underweight (BMI < 18.5). Evaluation of physical activity was conducted through the questions in the International Physical Activity Questionnaire, which graded activity into three degrees: low, moderate, and high.^7^ A questionnaire^8^ containing the usual dietary intake was completed by United Kingdom Biobank participants during the baseline assessment. The intake of fruits (fresh fruit intake and dried fruit intake), vegetables (cooked vegetable intake and salad/raw vegetable intake), fish (oily fish intake and non-oily fish intake), processed meat and unprocessed red meat (beef intake, lamb/mutton intake, and pork intake) from the United Kingdom Biobank food intake questionnaire was used to calculate the health diet scores (Wang et al., 2021): fruit intake ≥ three pieces per day, vegetable intake ≥ four tablespoons per day, fish intake ≥ twice per week, processed meat intake ≤ twice per week, unprocessed red meat intake ≤ twice per week. Each favorable dietary factor gave a point, so the healthy diet scores were 0–5. The serum concentrations of glycosylated hemoglobin and total cholesterol were regarded as continuous variables. Vascular problems included angina, heart attack, stroke, and high blood pressure.

### Data Analysis

All analyses were performed using R version 4.0.2. The data are summarized descriptively. Continuous variables are represented as mean (standard deviation) and comparison between the two groups was performed by independent sample *t* test. The classification variables are represented as percentages (%) and the rate was compared by χ^2^ test. The link between air pollution and hearing impairment was investigated using a multivariate logistic regression model with and without adjusting for other variables. Model 1 was unadjusted, Model 2 was adjusted for age and gender, and Model 3 was further adjusted for race, educational level, employment, smoking status and alcohol consumption frequency, BMI, physical activity, glycosylated hemoglobin, total cholesterol, and vascular diseases (heart attack, stroke, angina, and hypertension). Moreover, we evaluated the association between subgroups stratified by healthy diet scores (low: 0–2, and high: 3–5), gender (female and male) and age (≤50, 51–60, and >60). The Wald test was used to test interactions among subgroups. *P* < 0.05 (two-sided test) was considered statistically significant.

## Results

In total, 158,811 subjects were enrolled in this study, including 18,881 (11.9%) with hearing impairment and 139,930 (88.1%) with normal hearing, 54.5% were female (*n* = 86,516), 91.7% were white (*n* = 145,633), with the mean (standard deviation) age of 56.68 (8.15) years. The distribution of baseline characteristics and air pollution in the two groups is shown in Table 1. Except for physical activity, other variables were significantly distributed in the two groups (*P* < 0.05). In comparison to the group of people with normal hearing, the subjects in the hearing impairment group were older on average, non-whites. In addition, they were more likely to be obese and to have cardiovascular problems. Furthermore, the hearing impairment group was exposed to higher mean annual concentrations of air pollutants than the normal hearing group (Table 1).

**TABLE 1:** Characteristics of participants (*N* = 158,811).

	Normal hearing	Hearing impairment	*P*
*N*	139,930	18,881	
Age (years), mean (SD)	56.21 (8.13)	60.13 (7.40)	<0.001
Gender (%)			0.004
Female	76,414 (54.6)	10,102 (53.5)	
Male	63,516 (45.4)	8,779 (46.5)	
Race (%)			<0.001
White ethnicity	129,996 (93.2)	15,637 (83.3)	
Mixed ethnicity	1,053 (0.8)	137 (0.7)	
Asian ethnicity	3,541 (2.5)	1,328 (7.1)	
Black ethnicity	3,019 (2.2)	1,026 (5.5)	
Chinese ethnicity	462 (0.3)	123 (0.7)	
Other ethnicity	1,336 (1.0)	523 (2.8)	
Education (%)			<0.001
Other professional qualification	7,113 (5.9)	1,191 (8.6)	
College or university degree	48,983 (40.7)	5,098 (36.8)	
O level/GCSEs or equivalent	30,497 (25.4)	3,580 (25.9)	
CSEs or equivalent	8,280 (6.9)	845 (6.1)	
A/AS levels or equivalent	16,528 (13.7)	1,670 (12.1)	
NVQ or HND or HNC or equivalent	8,876 (7.4)	1,465 (10.6)	
Employment (%)			<0.001
Inpaid employment or self-employed	81,816 (59.0)	7,467 (40.1)	
Retired	44,969 (32.4)	9,136 (49.1)	
Looking after home and/or family	4,195 (3.0)	474 (2.5)	
Unable to work because of sickness or disability	3,575 (2.6)	883 (4.7)	
Unemployed	2,996 (2.2)	478 (2.6)	
Doing unpaid or voluntary work	678 (0.5)	115 (0.6)	
Full-time or part-time student	384 (0.3)	66 (0.4)	
BMI (%), kg/m^2^			<0.001
Underweight	721 (0.5)	121 (0.6)	
Normal weight	46,225 (33.2)	5,590 (30.0)	
Overweight	58,610 (42.1)	7,817 (41.9)	
Obesity	33,548 (24.1)	5,127 (27.5)	
Smoke (%)			<0.001
Never	77,310 (55.4)	10,140 (54.0)	
Previous	48,353 (34.7)	6,572 (35.0)	
Current	13,839 (9.9)	2,054 (10.9)	
Drink frequency (%)			<0.001
Daily or almost daily	29,132 (20.8)	3,327 (17.7)	
Three or four times a week	32,246 (23.1)	3,443 (18.3)	
Once or twice a week	35,588 (25.5)	4,367 (23.2)	
Occasional drinkers	32,275 (23.1)	5,095 (27.0)	
Never	10,551 (7.5)	2,609 (13.8)	
Physical activity (%)			0.061
Low	20,316 (17.6)	2,658 (18.1)	
Moderate	46,945 (40.7)	5,839 (39.7)	
High	48,013 (41.7)	6,198 (42.2)	
HbA1c, mean (SD), mmol/mol	36.02 (6.50)	37.55 (8.10)	<0.001
TC, mean (SD), mmol/L	5.71 (1.14)	5.60 (1.20)	<0.001
Vascular problems (%)			<0.001
None	10,0638 (73.8)	11,826 (64.9)	
Hypertension	29,376 (21.5)	4,868 (26.7)	
Heart attack, angina, or stroke	3,184 (2.3)	703 (3.9)	
High blood pressure and heart attack, angina, or stroke	3,235 (2.4)	813 (4.5)	
Air pollution			
PM_2.5_, mean (SD), μg/m^3^	9.88 (0.91)	9.94 (0.95)	<0.001
PM_10_, mean (SD), μg/m^3^	16.28 (1.82)	16.45 (1.85)	<0.001
PM_2.5_ absorbance, mean (SD), per-meter	1.21 (0.27)	1.24 (0.29)	<0.001
NO_X_, mean (SD), μg/m^3^	43.52 (14.44)	44.92 (15.83)	<0.001
NO_2_, mean (SD), μg/m^3^	26.88 (7.21)	27.72 (7.70)	<0.001

*Abbreviations: N, number; SD, standard deviation; GCSEs, general certificate of secondary educations; CSEs, certificate of secondary educations; NVQ, national vocational qualification; HND, higher national diploma; HNC, higher national certificate; BMI, body mass index; HbA1c, glycosylated hemoglobin; TC, total cholesterol; PM, particulate matter; NO_2_, nitrogen dioxides; NO_X_, nitrogen oxides.*

Table 2 shows the risks of several air pollutants and hearing impairment. Model 1 (without adjustment for any confounders) showed significant associations between air pollutants and hearing impairment (*P* < 0.001) [PM_2.5_: odds ratio (OR) = 2.03, 95% confidence interval (95% CI) 1.73–2.40; PM_10_: OR = 1.64, 95% CI 1.51–1.78; PM_2.5_ absorbance: OR = 1.48, 95% CI 1.40–1.56; NO_X_: OR = 1.06, 95% CI 1.05–1.07; NO_2_: OR = 1.17, 95% CI 1.41–1.19]. After adjusting for age and gender, Model 2 showed that air pollutants were still significantly associated with hearing impairment (*P* < 0.001), and all OR values were larger than Model 1 (PM_2.5_: OR = 3.02, 95% CI 2.56–3.57; PM_10_: OR = 1.82, 95% CI 1.67–1.97; PM_2.5_ absorbance: OR = 1.67, 95% CI 1.59–1.77; NO_X_: OR = 1.09, 95% CI 1.08–1.10; NO_2_: OR = 1.24, 95% CI 1.21–1.26). Except for PM_2.5_ and PM_2.5_ absorbance, which showed no significant associations with hearing impairment (*P* = 0.970 and *P* = 0.063, respectively), we observed that the associations between the other pollutants and hearing impairment remained in Model 3 after further adjusting for other confounders on the basis of Model 2 (PM_10_: OR = 1.15, 95% CI 1.02–1.30, *P* = 0.023; NOx: OR = 1.02, 95% CI 1.00–1.03, *P* = 0.040; NO_2_: OR = 1.03, 95% CI 1.01–1.06, *P* = 0.044), even though the estimates were lower than those in Models 1 and 2.

**TABLE 2:** Association of air pollution and hearing impairment.

	Model 1		Model 2		Model 3	
	OR (95% CI)	*P*	OR (95% CI)	*P*	OR (95% CI)	*P*
PM_2.5_	2.03 (1.73–2.40)	<0.001	3.02 (2.56–3.57)	<0.001	1.01 (0.79–1.29)	0.970
PM_10_	1.64 (1.51–1.78)	<0.001	1.82 (1.67–1.97)	<0.001	1.15 (1.02–1.30)	0.023
PM_2.5_ absorbance	1.48 (1.40–1.56)	<0.001	1.67 (1.59–1.77)	<0.001	1.08 (0.99–1.18)	0.063
NO_X_	1.06 (1.05–1.07)	<0.001	1.09 (1.08–1.10)	<0.001	1.02 (1.001–1.03)	0.040
NO_2_	1.17 (1.14–1.19)	<0.001	1.24 (1.21–1.26)	<0.001	1.03 (1.01–1.06)	0.044

*Abbreviations: OR, odds ratio; CI, confidence interval; PM, particulate matter; NO_2_, nitrogen dioxides; NO_X_, nitrogen oxides.*

*Model 1: unadjusted.*

*Model 2: adjusted for age and gender.*

*Model 3: adjusted for age, gender, race, education, employment, smoking, drink frequency, body mass index, physical activity, glycosylated hemoglobin, total cholesterol, and vascular disease (heart attack, stroke, angina, and hypertension).*

Table 3 shows the associations between several air pollutants and hearing impairment, stratified by healthy diet scores. In this study, no significant associations and moderating effects were observed. After stratification by age (Table 4), we found that PM_10_, PM_2.5_ absorbance, NO_X_, and NO_2_ were associated with hearing impairment in participants up to and including 50 years of age (PM_10_: OR = 1.62, 95% CI 1.20–2.18, *P* = 0.002; PM_2.5_ absorbance: OR = 1.32, 95% CI 1.08–1.61, *P* = 0.006; NO_X_: OR = 1.04, 95% CI 1.01–1.08, *P* = 0.014; NO_2_: OR = 1.09, 95% CI 1.01–1.17, *P* = 0.031). In participants aged 51 to 60 years and above 60, there was no connection between air pollution and hearing impairment. Additionally, there was a statistically significant interaction between age and air pollution with hearing impairment (*P* < 0.05). Further, after stratifying by gender (Table 5), we found that NO_X_ and NO_2_ were correlated with hearing impairment in men.

**TABLE 3:** Associations of air pollution and hearing impairment in subgroups stratified by healthy diet scores.

	Low (*N* = 58324)		High (*N* = 94414)		*P*-interaction
	OR (95% CI)	*P*	OR (95% CI)	*P*	
PM_2.5_	1.05 (0.69, 1.61)	0.823	0.84 (0.62, 1.14)	0.271	0.403
PM_10_	1.20 (0.97, 1.49)	0.086	1.14 (0.98, 1.32)	0.094	0.688
PM_2.5_ absorbance	1.10 (0.95, 1.27)	0.191	1.10 (0.99, 1.22)	0.060	0.902
NO_X_	1.02 (0.99, 1.05)	0.147	1.01 (0.99, 1.03)	0.498	0.300
NO_2_	1.04 (0.99, 1.10)	0.161	1.02 (0.98, 1.06)	0.315	0.377

*Abbreviations: OR, odds ratio; CI, confidence interval; PM, particulate matter; NO_2_, nitrogen dioxides; NO_X_, nitrogen oxides.*

*All models were adjusted for age, gender, race, education, employment, smoking, drink frequency, body mass index, physical activity, glycosylated hemoglobin, total cholesterol, and vascular disease (heart attack, stroke, angina, and hypertension).*

*This subgroup included 152,738 participants because of the missing data of dietary information for 6,073 participants.*

**TABLE 4:** Associations of air pollution and hearing impairment in subgroups stratified by age.

	≤50 (*N* = 40,978)		51–60 (*N* = 53,844)		>60 (*N* = 63,989)		*P*-interaction
	OR (95% CI)	*P*	OR (95% CI)	*P*	OR (95% CI)	*P*	
PM_2.5_	1.74 (0.96–3.14)	0.067	1.02 (0.66–1.59)	0.918	0.80 (0.57–1.13)	0.198	0.013
PM_10_	1.62 (1.20–2.18)	0.002	1.15 (0.92–1.43)	0.215	1.04 (0.88–1.23)	0.647	0.005
PM_2.5_ absorbance	1.32 (1.08, 1.61)	0.006	1.04 (0.89–1.21)	0.626	1.05 (0.93–1.18)	0.417	0.029
NO_X_	1.04 (1.01–1.08)	0.014	1.02 (0.99–1.05)	0.247	1.00 (0.98–1.02)	0.921	0.005
NO_2_	1.09 (1.01–1.17)	0.031	1.03 (0.97–1.09)	0.292	1.01 (0.97–1.06)	0.641	0.017

*Abbreviations: OR, odds ratio; CI, confidence interval; PM, particulate matter; NO_2_, nitrogen dioxides; NO_X_, nitrogen oxides.*

*All models were adjusted for gender, race, education, employment, smoking, drink frequency, body mass index, physical activity, glycosylated hemoglobin, total cholesterol, and vascular disease (heart attack, stroke, angina, and hypertension).*

**TABLE 5:** Associations of air pollution and hearing impairment in subgroups stratified by gender.

	Female (*N* = 86516)		Male (*N* = 72295)		*P*-interaction
			
	OR (95% CI)	*P*	OR (95% CI)	*P*	
PM_2.5_	0.86 (0.61–1.22)	0.406	1.19 (0.83–1.69)	0.346	0.191
PM_10_	1.16 (0.98–1.38)	0.085	1.15 (0.97–1.37)	0.118	0.914
PM_2.5_ absorbance	1.10 (0.98–1.23)	0.112	1.07 (0.95–1.21)	0.261	0.920
NO_X_	1.01 (0.98–1.02)	0.879	1.03 (1.01–1.05)	0.011	0.061
NO_2_	1.02 (0.97–1.06)	0.489	1.05 (1.00–1.09)	0.049	0.216

*Abbreviations: OR, odds ratio; CI, confidence interval; PM, particulate matter; NO_2_, nitrogen dioxides; NO_X_, nitrogen oxides.*

*All models were adjusted for age, race, education, employment, smoking, drink frequency, body mass index, physical activity, glycosylated hemoglobin, total cholesterol, and vascular disease (heart attack, stroke, angina, and hypertension).*

## Discussion

In this cross-sectional study, we investigated the association between hearing impairment and air pollution (comprising PM_2.5_, PM_10_, PM_2.5_ absorbance, NO_X_, and NO_2_) using United Kingdom Biobank data. We found that exposure to PM_10_, NO_X_, and NO_2_ was linked to hearing impairment after adjusting for confounding factors, while PM_2.5_ and PM_2.5_ absorbance showed no similar correlations. Furthermore, there was no modification of these associations by a healthy diet. Regarding age, interaction effects were observed.

The relationship between air pollution and hearing impairment has not been fully established yet. Several studies indicated that exposure to NO_2_ could be related to hearing problems. Chang et al. (2020) found that people exposed to moderate (hazard ratio, HR = 1.40, 95% CI 1.27–1.54) and high levels of NO_2_ (HR = 1.63, 95% CI 1.48–1.81) were at higher risk of developing sensorineural hearing loss than those exposed to the low level. The results of Tsai et al. (2020) were similar, finding a significantly increased risk of SSNHL in those exposed to high concentrations of NO_2_ (adjusted HR = 1.02, 95% CI 1.01–1.04). Likewise, Choi et al. (2019) discovered that SSNHL was associated with short-term exposure to NO_2_ (14 days) (adjusted OR = 3.12, 95% CI 2.16–4.49). Consistent with previous studies, NO_2_ was associated with hearing impairment in our study. Moreover, NO_X_, a term that contains several nitrogen compounds but is mainly composed of nitrogen oxide and NO_2_, showed an association with hearing impairment.

In contrast to our expectations, we found a significant association between PM_10_ and hearing impairment but not PM_2.5_. Conversely, previous studies (Choi et al., 2019; Lee et al., 2019) showed no correlation between PM_10_ and hearing impairment. A study reported (Tsai et al., 2020) a significantly higher risk of developing SSNHL with moderate (adjusted HR = 1.58, 95% CI 1.21–2.06) or high (adjusted HR = 1.32, 95% CI 1.00–1.74) level exposure to PM_2.5_ compared to those exposed to the low level. And another study discovered a slight negative association between the maximum PM_2.5_ concentration and the admission rate of SSNHL (Lee et al., 2019). In 2017, a study (Strak et al., 2017) in a large national health survey reported that oxidative potential of PM_2.5_ rather than PM_2.5_, was associated with diabetes prevalence, indicating that the impact of particulate matter on diabetes might vary with the compositions. According to a study (Yin and Harrison, 2008) conducted at three sites (urban roadside, central urban background, and rural) in Birmingham, United Kingdom, organics, nitrate, and sulfate accounted for a substantial amount of the overall mass for both PM_10_ and PM_2.5_. This research also showed that proportions of these three major parts and other secondary compositions like iron-rich dust and sodium chloride varied in both. Although discrepancies in associations with diseases after PM_2.5_ and PM_10_ exposure could be explained by different compositions of particulate matter, the evidence may still be limited. More research is required to clarify this issue in the future.

Oxidative stress and mitochondrial dysfunction play a crucial role in hearing impairment (Yamasoba et al., 2013). Air pollution might be involved in oxidative stress by producing or directly acting as reactive oxygen species (Kelly, 2003), which can then induce mitochondrial damage (Rodríguez-Martínez et al., 2013). Dysfunctional mitochondria increase reactive oxygen species generation and accumulation, reducing the mitochondrial membrane potential, activating the apoptosis pathway, and causing the death of inner ear hair cells (Park et al., 2016). What’s more, air pollution might indirectly be associated with hearing impairment by causing cardiovascular diseases through pro-inflammatory pathways and the production of reactive oxygen species (Simkhovich et al., 2008; Brook et al., 2010). It has been demonstrated that cardiovascular diseases are risk factors for hearing impairment (Oron et al., 2014; Tan et al., 2018). Nonetheless, the link between air pollution and hearing impairment was still evident after adjusting for related vascular problems in Model 3, suggesting that other mechanisms may also be involved in the link between air pollution and hearing impairment.

There was evidence that a healthy diet could protect against hearing impairment by reducing vascular damage, decreasing inflammation, and inhibiting oxidative damage (Curhan et al., 2020; Yevenes-Briones et al., 2021). Based on similar mechanistic pathways, modifying the health effects of air pollution by diet may be possible. But in our study, no effect modification of diet was observed. Studies previously showed an interaction between dietary patterns and air pollution exposure on health-related outcomes. In a birth cohort in Northeast China, animal foods pattern was found to significantly modify the association between exposure to NO_2_ and carbon monoxide and gestational diabetes mellitus, with higher intake related to a higher rate of gestational diabetes mellitus following exposure to air pollution (Hehua et al., 2021). A Mediterranean diet reduced cardiovascular disease mortality risk related to long-term exposure to air pollutants in a large prospective US cohort (Lim et al., 2019). A prospective cohort study of Chinese older adults reported that a plant-based dietary pattern mitigated the adverse effects of air pollution on cognitive function (Zhu et al., 2022).

It seems to be accepted that hearing impairment becomes more common with increasing age (Díaz et al., 2016). Nevertheless, the association between air pollution and hearing impairment was only found in participants younger under or equal to 50 years of age in this study. An interaction effect between age and air pollution on hearing impairment was also observed. Age is an unmodifiable risk factor for hearing impairment, which could lead to cochlear aging (Yamasoba et al., 2013). However, modifiable risk factors play a significant part in the development of hearing impairment at a relatively young age (i.e., <85 years old), while their effects decrease in the oldest people (i.e., ≥85 years old) (Zhan et al., 2010). Therefore, we speculated that air pollution, a modifiable risk factor, might have a greater impact on people younger than or equal to 50 years old compared to those over 50 years old, even if our study subjects were all under 85 years old.

Our research used data from the United Kingdom Biobank, a national cohort with good quality control. Additionally, the hearing test was based on the DTT data in the United Kingdom Biobank, which represented an ecologically effective and objective hearing indicator. We also adjusted for many confounders (including demographic information, lifestyle, and related diseases affecting hearing) to reduce their potential impact. However, our research also had some limitations. Above all, the cross-sectional design of this study was inadequate to account for the cause and effect between air pollution and hearing impairment, and further longitudinal studies are needed. Second, the sample of participants in United Kingdom Biobank was suggested to be unrepresentative of the general population because of the bias toward recruiting participants who were generally healthier and had a higher socioeconomic status (Fry et al., 2017). Hence, the subsample from United Kingdom Biobank and estimated hearing impairment rate in this study might not be representative of the general population. Third, like other epidemiological studies of air pollution, there might be potential misclassifications of air pollution exposure in this study because air pollution exposure was evaluated at the place of residence. Fourth, in the United Kingdom, where emissions regulations are strict and average pollution level is relatively low, it is not clear to what extent this study can be generalizable to other settings. Finally, in spite of adjusting for many confounders in our study, the potential effects of residual confounds of unmeasured variables could not be excluded, such as the use of ototoxic drugs, which was not considered due to lack of data.

## Conclusion

In conclusion, we found that exposure to PM_10_, NO_X_, and NO_2_ was associated with hearing impairment in British adults, while PM_2.5_ and PM_2.5_ absorbance did not show similar correlations. Our findings may help researchers pay more attention to the impact of air pollution on hearing impairment and provide a basis for developing effective prevention strategies.

## Data Availability Statement

The data supporting the results of this study can be found in the website of UK Biobank (www.ukbiobank.ac.uk) upon application.

## Ethics Statement

The study involving human participants was carried out with the ethical approval obtained by United Kingdom Biobank from the National Health Service National Research Ethics Service.

## Author Contributions

YS, YT, and LY conceived the overall project and developed the methods as well as procedures throughout the study. DL and LY managed the data collection and data entry and carried out data verification and statistical analyses. LY drafted the first version of the manuscript. All authors oversaw statistical analysis, involved in the interpretation of the results, reviewed, and approved the final manuscript.

## Conflict of Interest

The authors declare that the research was conducted in the absence of any commercial or financial relationships that could be construed as a potential conflict of interest.

## Publisher’s Note

All claims expressed in this article are solely those of the authors and do not necessarily represent those of their affiliated organizations, or those of the publisher, the editors and the reviewers. Any product that may be evaluated in this article, or claim that may be made by its manufacturer, is not guaranteed or endorsed by the publisher.

## Footnotes

1
www.UKbiobank.ac.UK

2
https://biobank.ctsu.ox.ac.UK/crystal/label.cgi?id=100049

3
http://www.sahsu.org/

4
http://www.bioshare.eu/

5
http://www.escapeproject.eu/

6
https://biobank.ndph.ox.ac.uk/showcase/ukb/docs/EnviroExposEst.pdf

7
https://biobank.ndph.ox.ac.uk/showcase/ukb/docs/ipaq_analysis.pdf

8
https://biobank.ndph.ox.ac.uk/showcase/label.cgi?id=100052
